# Causes of death in the Taabo health and demographic surveillance system, Côte d'Ivoire, from 2009 to 2011

**DOI:** 10.3402/gha.v8.27271

**Published:** 2015-05-08

**Authors:** Siaka Koné, Thomas Fürst, Fabienne N. Jaeger, Emmanuel L. J. C. Esso, Nahoua Baïkoro, Kouamé A. Kouadio, Lukas G. Adiossan, Fabien Zouzou, Louis I. Boti, Marcel Tanner, Jürg Utzinger, Bassirou Bonfoh, Daouda Dao, Eliézer K. N'Goran

**Affiliations:** 1 Centre Suisse de Recherches Scientifiques en Côte d'Ivoire, Abidjan, Côte d'Ivoire; 2 Department of Epidemiology and Public Health, Swiss Tropical and Public Health Institute, Basel, Switzerland; 3 University of Basel, Basel, Switzerland; 4 Centre for Health Policy, Imperial College London, London, United Kingdom; 5 Department of Infectious Disease Epidemiology, Imperial College London, London, United Kingdom; 6 Hôpital Générale de Taabo, Taabo Cité, Côte d'Ivoire; 7 Fairmed, Bern, Switzerland; 8 Unité de Formation et de Recherche Biosciences, Université Félix Houphouët-Boigny, Abidjan, Côte d'Ivoire

**Keywords:** mortality, cause of death, verbal autopsy, InterVA-4, health and demographic surveillance system, Côte d'Ivoire

## Abstract

**Background:**

Current vital statistics from governmental institutions in Côte d'Ivoire are incomplete. This problem is particularly notable for remote rural areas that have limited access to the health system.

**Objective:**

To record all deaths from 2009 to 2011 and to identify the leading causes of death in the Taabo health and demographic surveillance system (HDSS) in south-central Côte d'Ivoire.

**Design:**

Deaths recorded in the first 3 years of operation of the Taabo HDSS were investigated by verbal autopsy (VA), using the InterVA-4 model. InterVA-4 is based on the World Health Organization 2012 VA tool in terms of input indicators and categories of causes of death.

**Results:**

Overall, 948 deaths were recorded, of which 236 (24.9%) had incomplete VA data. Among the 712 deaths analyzed, communicable diseases represented the leading causes (58.9%), with most deaths attributed to malaria (*n*=129), acute respiratory tract infections (*n*=110), HIV/AIDS (*n*=80), and pulmonary tuberculosis (*n*=46). Non-communicable diseases accounted for 18.9% of the deaths and included mainly acute abdomen (*n*=38), unspecified cardiac diseases (*n*=15), and digestive neoplasms (*n*=13). Maternal and neonatal conditions accounted for 8.3% of deaths, primarily pneumonia (*n*=19) and birth asphyxia (*n*=16) in newborns. Among the 3.8% of deaths linked to trauma and injury, the main causes were assault (*n*=6), accidental drowning (*n*=4), contact with venomous plants/animals (*n*=4), and traffic-related accidents (*n*=4). No clear causes were determined in 10.0% of the analyzed deaths.

**Conclusions:**

Communicable diseases remain the predominant cause of death in rural Côte d'Ivoire. Based on these findings, measures are now being implemented in the Taabo HDSS. It will be interesting to monitor patterns of mortality and causes of death in the face of rapid demographic and epidemiological transitions in this part of West Africa.

A key issue in the development of reliable population statistics in resource-constrained settings is the establishment of a robust registration policy for deaths and the underlying causes. However, little is known about the mortality and causes of death in remote areas in large parts of sub-Saharan Africa (1). Indeed, still today, many Africans ‘are born and die without leaving a trace in any legal record or official statistic’ (2). In 2001, for example, 42 out of 47 countries listed from sub-Saharan Africa were unable to provide any national data on causes of death (1). Over the past 15 years, civil registration and vital statistics gained attention from researchers, development partners, and policy makers (3).

Côte d'Ivoire is among the countries with poor vital statistics. According to the World Health Statistics 2010 report, put forth by the World Health Organization (WHO), coverage of death registration in Côte d'Ivoire between 2000 and 2008 was below 25% (4). Subsequent World Health Statistics reports did not mention any coverage estimates (5–8). The political and military crisis, which started in the late 1990s and lasted until 2011, repeatedly shattered the country, weakened the health system, and also hampered birth registration in Côte d'Ivoire. According to the most recent Multiple Indicator Cluster Survey (MICS), carried out in 2011–2012, only 65% of all children under the age of 5 years were registered and birth registration was considerably higher in children aged 15–17 years (87%) (9). The survey also revealed considerable differences between the birth registration rates in urban (90%) and rural areas (66%) among children below the age of 18 years (9). The World Health Statistics from 2010 to 2014 confirmed unsatisfactory birth registration coverage, with estimates ranging between 55 and 65% (4–8). Of note, Côte d'Ivoire has gone through several rounds of demographic data collection: two national population censuses (1988 and 1998), Demographic and Health Surveys (1994 and 1998–1999), and MICSs (2001, 2006, and 2011–2012). These surveys usually collected information on child mortality, maternal health, HIV/AIDS, malaria, and other diseases. However, most of these estimates are dated, and the most recent MICS (2011–2012) did not determine detailed causes of death (9). Furthermore, the prior surveys have limitations such as discrimination against certain groups in the specification of causes of death. Moreover, causes of death are often only reported from hospitals, where the diagnostic equipment allows for at least some precision in the certification (10). It follows that the data are not representative of the general population. Indeed, the data are inadequate for many African countries, particularly for rural settings, where most deaths occur in the community, outside of the health system, thus without any registration and autopsy. As a result, death registration is fragmented and provides only patchy information on the age of individuals at death (11, 12) or the underlying causes in the general population.

In the present study, we describe the main causes of death that occurred between 2009 and 2011 in the Taabo health and demographic surveillance system (HDSS), located in south-central Côte d'Ivoire (13). Information on the sex- and age-specific causes of death were determined by verbal autopsy (VA) and provide important insights into the epidemiological and public health situation in this cohort of about 40,000 mainly rural dwellers in Côte d'Ivoire. These insights may not only help better address and reduce preventable deaths in the surveyed communities, but they may also help mitigate negative health impacts in other rural communities throughout the West African region.

## Methods

### Taabo HDSS and study population

The Taabo HDSS was established in 2008 and the initial census revealed a population of 37,792. In December 2011 the population was 39,422, and in December 2013 it had reached 42,480 (13). The Taabo HDSS includes one small town (Taabo Cité, 7,514 inhabitants at the end of 2013), 13 main villages, and over 100 hamlets. The establishment of the Taabo HDSS, the longitudinal surveillance of demographic and health data, and the implementation of specific interventions and research projects were approved by the institutional research commissions of the Centre Suisse de Recherches Scientifiques en Côte d'Ivoire (Abidjan, Côte d'Ivoire) and the Swiss Tropical and Public Health Institute (Basel, Switzerland). Ethical clearance was obtained from the ethics committees in Côte d'Ivoire (reference no. 1086 MSHD/CNEF) and Basel (EKBB, reference no. 316/08).

All households belonging to the Taabo HDSS are visited three times a year by trained field enumerators. They conduct demographic surveillance, including the registration and monitoring of migration, pregnancies, births, and deaths. Monitoring pregnancies helps enumerators obtain information on stillbirths, abortions, and neonatal deaths. All deaths of permanent residents are recorded and – as with other HDSS sites – whenever possible examined by VA techniques in order to determine the most likely causes of death (14–19). All applied registration and monitoring methods were developed and standardized in order to ensure that the data collected are of high quality and will allow for cross-site comparison (10, 20–23). As a member of the International Network for the Continuous Demographic Evaluation of Populations and Their Health (INDEPTH; http://indepth-network.org), the Taabo HDSS adheres to INDEPTH's standards. Further details regarding the Taabo HDSS have been published elsewhere (13).

### VA procedure in the Taabo HDSS

The reporting of death is facilitated by key informants in the communities who observe and record any death occurring in the study area. The information is then transmitted to a Taabo HDSS VA supervisor or a field enumerator who informs a VA supervisor. Deaths that might have been missed by the key informants can subsequently be identified during demographic surveillance rounds. Whenever possible, the VA supervisor visits the household in which the death has occurred within 2 weeks and contacts the Taabo HDSS data center for verification of the event. Once it has been verified that the deceased was an HDSS resident, the VA supervisor completes a standardized VA questionnaire with one of the deceased's close relatives.

Initially, completed VA questionnaires were submitted to two physicians who independently determined the direct and underlying cause of death and coded it according to the WHO International Classification of Diseases, version 10 (ICD-10) (24). However, this practice proved relatively slow, somewhat expensive, and there was considerable inter-observer variation regarding the causes of death. Since 2012, the computer automated InterVA-4 model has been validated and admitted for use in research and civil registration, both within already enumerated populations and also as a stand-alone death registration tool (25–28). For the present analysis, the InterVA-4 model was employed.

### VA data interpretation with InterVA-4 to derive causes of death

The InterVA-4 tool is a freely available standard computerization model for interpreting VA data and determining causes of death. It has been designed to use the VA input indicators defined in the 2012 WHO VA instrument and to deliver causes of death compatible with the ICD-10. The causes of death are categorized into 62 groups, as defined in the 2012 WHO VA instrument (25–28).

The InterVA-4 model was developed on the basis of Bayes’ theorem and is therefore an application of the Bayesian approach for diagnostic help (25–29). If the event of interest (*A*) depends on different mutually exclusive causes *C*_1_, *C*_2_, …, *C*_*m*_ (for instance, causes of death) and other factors *S*_1_, *S*_2_, …, *S*_*n*_ (for instance, different signs and symptoms leading to death), then Bayes’ general theorem for each *C*_*i*_ and *S*_*j*_ can be stated as

$$\text{P}(C_{i}|S_{j})=\frac{\text{P}(S_{j}|C_{i})\times \text{P}(C_{i})}{\text{P}(S_{j}|C_{i})\times \text{P}(C_{i})+\text{P}(S_{j}|!C_{i})\times \text{P}(!C_{i})}$$

*with* P(!*C_i_*) = (1−P(*C_i_*)).

For the complete set of causes of death *C*_1_, *C*_2_, …, *C*_*m*_, a set of probabilities for each *C*_*i*_ can be computed using a normalizing assumption so that the total conditional probability of all causes sums up to unity:

$$\text{P}(C_{i}|S_{j})=\frac{\text{P}(S_{j}|C_{i})\times \text{P}(C_{i})}{\sum_{i=1}^{m}\text{P}\left(C_{i}\right)}$$

While using an initial set of unconditional probabilities for the causes of death *C*_1_, *C*_2_, …, *C*_*m*_(P(*C*_*i*_∣*S*_0_)) and the matrix of the conditional probabilities P(*S*_*j*_∣*C*_*i*_) for indicators *S*_1_, *S*_2_, …, *S*_*n*_ and causes of death *C*_1_, *C*_2_, …, *C*_*m*_, it is possible to apply the same calculation for each *S*_1_, *S*_2_, …, *S*_*n*_ that applies to one particular death:

$$\text{P}(C_{i}|S_{S_{j\ldots n}})=\frac{\text{P}(S_{j}|C_{i})\times \text{P}(C_{i}|S_{0\ldots n-1})}{\sum_{i=1}^{m}\text{P}\left(C_{i}|S_{0\ldots n-1}\right)}$$

In short, with the exception of a minority of interviews where information is contradictory or inconsistent and the cause of death has to be classified as undetermined despite a completed VA interview, usually up to a maximum of three most likely causes of death and their probabilities are estimated per case. Whenever these causes do not add up to 100%, the remaining percentage is classified as undetermined. Because we are working with probabilities, one death can thus contribute to the statistics for several possible causes of death, but never at more than 100%. These idiosyncrasies of InterVA-4 lead to minor rounding errors and explain small differences between the disaggregated data and the summed up totals as presented in this study. Like its predecessor version, InterVA-4 employs special procedures for HIV/AIDS and malaria, because these diseases vary greatly from one setting to another (26). For malaria, unconditional probability is applied to the causes of death, especially due to sickle cell disease as there is a close link between these two conditions (26, 30). For the present analysis, all identified causes of death were aggregated into 14 broad groups, as predefined by INDEPTH, using the statistical software package STATA version 12 (StataCorp, College Station, TX, USA).

## Results

### Number of deaths recorded in 2009–2011

The sex- and age-specific distribution of the study population, crude numbers of deaths, and mortality rates per 1,000 person-years are presented in Table 1. Overall, 948 deaths were reported among all people registered in the Taabo HDSS between 2009 and 2011. The overall mortality rate was 9.1 deaths per 1,000 person-years, with a slightly higher rate in males compared to females (9.5 *vs*. 8.8 deaths per 1,000 person-years). Rates peaked for both sexes in the youngest (i.e. <1 year, 123.2 and 130.0 deaths per 1,000 person-years for males and females, respectively) and the oldest age groups (i.e. ≥65 years, 61.4 and 45.7 deaths per 1,000 person-years for males and females, respectively).

**Table 1:** Person-year structure, total number of deaths, and mortality rates by sex and age in the Taabo HDSS, 2009–2011

	Male			Female			Total*		
	
Age (years)	_a_PY_x_	_a_D_x_	_a_M_x (‰)_	_a_PY_x_	_a_D_x_	_a_M_x (‰)_	_a_PY_x_	_a_D_x_	_a_M_x (‰)_
<1	706	87	123.2	700	91	130.0	1,406	178	126.6
1–4	6,476	100	15.4	6,475	97	15.0	12,951	197	15.2
5–14	16,198	28	1.7	14,769	27	1.8	30,967	55	1.8
15–49	25,617	139	5.4	22,869	110	4.8	48,485	249	5.1
50–64	3,792	62	16.4	3,176	37	11.6	6,967	99	14.2
≥65	1,595	98	61.4	1,577	72	45.7	3,173	170	53.6
All*	54,384	514	9.5	49,566	434	8.8	103,949	948	9.1

_a_PY_x_=total person-years lived in the sex-specific cohort from age a to age x.

_a_D_x_=total number of deaths in the sex-specific cohort from age a to age x.

_a_M_x (‰)_=total mortality rate per 1,000 person-years in the sex-specific cohort from age a to age x.

* Idiosyncrasies of InterVA-4 lead to minor rounding errors and explain small differences between the disaggregated data and the summed up totals as presented in this study.

**Fig. 1:**
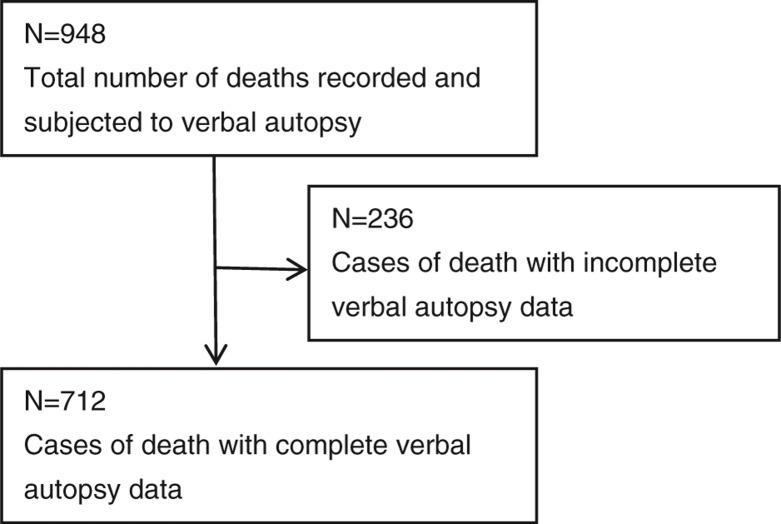
Flow chart detailing the operational result of verbal autopsies in the Taabo HDSS, 2009–2011. The total number of deaths recorded, reasons for exclusion from the present analysis, and the final study sample with complete verbal autopsy data are highlighted.

Of the 948 reported deaths, 236 (24.9%) had incomplete VA data. The two main reasons why such a considerable number of deaths could not be investigated appropriately were 1) the difficulty of finding a relative who could provide the information needed; and 2) the refusal to participate in or complete a VA interview. The remaining 712 deaths (75.1% of all reported deaths in the first 3 years of operation of the Taabo HDSS) were subjected to detailed InterVA-4 analysis and, hence, are considered as the basic total sample size for all subsequent analyses (Fig. 1). Age- and sex-specific mortality rates for the 236 deaths that could not be fully analyzed by InterVA-4 are summarized in Table 2.

**Table 2:** Deaths with no or incomplete verbal autopsy data by sex and age group in the Taabo HDSS, 2009–2011

	Male			Female			Total*		
	
Age (years)	_a_D_x_	%	_a_M_x__(‰)_	_a_D_x_	%	_a_M_x__(‰)_	_a_D_x_	%	_a_M_x__(‰)_
<1	13	1.4	18.4	16	1.7	22.9	29	3.1	20.6
1–4	27	2.8	4.2	26	2.7	4.0	53	5.6	4.1
5–14	7	0.7	0.4	4	0.4	0.3	11	1.2	0.4
15–49	45	4.7	1.8	32	3.4	1.4	77	8.1	1.6
50–64	21	2.2	5.5	7	0.7	2.2	28	3.0	4.0
≥65	15	1.6	9.4	23	2.4	14.6	38	4.0	12.0
All*	128	13.5	2.4	108	11.4	2.2	236	24.9	2.3

This table summarizes the cases of death that were registered in the Taabo HDSS but that could not or could only incompletely be investigated by means of verbal autopsy. Hence, these cases of death could not be included in the following causes of death analyses.

_a_D_x_=total number of deaths in the sex-specific cohort from age a to age x.

% = percentage of all 948 cases of death that were registered in the Taabo HDSS.

_a_M_x (‰)_=total mortality rate per 1,000 person-years in the sex-specific cohort from age a to age x.

* Idiosyncrasies of InterVA-4 lead to minor rounding errors and explain small differences between the disaggregated data and the summed up totals as presented in this study.

### Mortality due to communicable diseases by sex and age group

Communicable diseases represented the most frequent cause of death in the Taabo HDSS. Overall, 58.9% of the deaths subjected to InterVA-4 were due to communicable diseases, resulting in a mortality rate of 4.0 deaths per 1,000 person-years. Within the communicable disease cluster, malaria (18.0% of all examined deaths; 1.2 deaths per 1,000 person-years), acute respiratory tract infections (15.4% of all examined deaths; 1.1 deaths per 1,000 person-years), HIV/AIDS (11.2% of all examined deaths; 0.8 deaths per 1,000 person-years), and pulmonary tuberculosis (6.5% of all examined deaths; 0.4 deaths per 1,000 person-years) were the most frequently identified causes of death (Table 3).

**Table 3:** Mortality due to communicable diseases by sex and age group in the Taabo HDSS, 2009–2011

	<1 year			1–4 years			5–14 years			15–49 years			50–64 years			≥65 years		
	
Cause of death	_a_D_x_	%	_a_M_x__(‰)_	_a_D_x_	%	_a_M_x__(‰)_	_a_D_x_	%	_a_M_x__(‰)_	_a_D_x_	%	_a_M_x__(‰)_	_a_D_x_	%	_a_M_x__(‰)_	_a_D_x_	%	_a_M_x__(‰)_
	Male																	
Communicable diseases*	34	4.8	48.6	60	8.4	9.3	12	1.6	0.7	53	7.5	2.1	19	2.7	5.1	37	5.2	23.3
Acute respiratory infection, including pneumonia	16	2.3	22.8	10	1.5	1.6	3	0.4	0.2	12	1.6	0.5	8	1.1	2.1	13	1.8	8.2
Diarrheal diseases	3	0.4	3.9	5	0.7	0.8	0	0.0	0.0	0	0.0	0.0	0	0.0	0.0	1	0.1	0.3
HIV/AIDS-related death	5	0.7	7.1	8	1.1	1.2	4	0.5	0.2	9	1.3	0.4	4	0.5	1.0	2	0.3	1.3
Malaria	8	1.1	11.5	33	4.6	5.0	4	0.5	0.2	9	1.3	0.4	2	0.3	0.5	5	0.7	3.0
Measles	1	0.1	0.8	0	0.0	0.0	0	0.0	0.0	0	0.0	0.0	0	0.0	0.0	0	0.0	0.0
Meningitis and encephalitis	2	0.3	2.6	3	0.4	0.4	0	0.0	0.0	3	0.4	0.1	1	0.1	0.3	0	0.0	0.0
Other and unspecified infectious disease	0	0.0	0.0	1	0.1	0.2	0	0.0	0.0	4	0.6	0.2	1	0.1	0.3	4	0.5	2.3
Pulmonary tuberculosis	0	0.0	0.0	0	0.0	0.0	1	0.1	0.1	16	2.3	0.6	4	0.5	0.9	12	1.7	7.4
Sepsis (non-obstetric)	0	0.0	0.0	0	0.0	0.0	0	0.0	0.0	0	0.0	0.0	0	0.0	0.0	1	0.2	0.7
Tetanus	0	0.0	0.0	0	0.0	0.0	0	0.0	0.0	0	0.0	0.0	0	0.0	0.0	0	0.0	0.0
	Female																	
Communicable diseases*	44	6.1	62.5	58	8.1	9.0	16	2.2	1.1	49	6.9	2.1	14	1.9	4.3	23	3.1	14.3
Acute respiratory infection, including pneumonia	14	1.8	19.5	9	1.2	1.4	5	0.7	0.3	5	0.6	0.2	5	0.7	1.6	10	1.4	6.6
Diarrheal diseases	4	0.6	5.7	4	0.5	0.6	0	0.0	0.0	3	0.4	0.1	1	0.1	0.3	1	0.1	0.5
HIV/AIDS-related death	5	0.7	7.4	12	1.6	1.9	4	0.6	0.3	20	2.8	0.9	3	0.5	1.1	3	0.4	1.9
Malaria	15	2.1	20.9	31	4.2	4.7	5	0.6	0.3	13	1.9	0.6	1	0.1	0.3	4	0.5	2.3
Measles	0	0.0	0.0	1	0.1	0.1	0	0.0	0.0	0	0.0	0.0	0	0.0	0.0	0	0.0	0.0
Meningitis and encephalitis	4	0.6	6.3	1	0.1	0.2	0	0.0	0.0	0	0.0	0.0	0	0.0	0.0	0	0.0	0.0
Other and unspecified infectious disease	0	0.0	0.0	1	0.1	0.1	1	0.1	0.0	2	0.3	0.1	0	0.0	0.0	1	0.1	0.6
Pulmonary tuberculosis	0	0.0	0.0	0	0.0	0.0	1	0.2	0.1	5	0.7	0.2	3	0.4	0.9	4	0.5	2.2
Sepsis (non-obstetric)	1	0.1	1.2	1	0.1	0.1	0	0.0	0.0	1	0.1	0.0	0	0.0	0.0	0	0.0	0.0
Tetanus	1	0.1	1.4	0	0.0	0.0	0	0.0	0.0	0	0.0	0.0	0	0.0	0.0	0	0.0	0.0

_a_D_x_=cause-specific number of deaths in the sex-specific cohort from age a to age x.

% = percentage of all 712 cases of death that could be examined with the InterVA-4 tool.

_a_M_x (‰)_=cause-specific mortality rate per 1,000 person-years in the sex-specific cohort from age a to age x.

* Idiosyncrasies of InterVA-4 lead to minor rounding errors and explain small differences between the disaggregated data and the summed up totals as presented in this study.

Acute respiratory tract infections and malaria are the two most frequent causes of death among males (1.1 and 1.1 deaths per 1,000 male person-years) and females (1.0 and 1.4 deaths per 1,000 female person-years). The third most frequent cause of death was tuberculosis among males (0.6 deaths per 1,000 male person-years) and HIV/AIDS-related deaths among females (1.0 deaths per 1,000 female person-years). Acute respiratory tract infections, malaria, and HIV/AIDS ranked among the most important causes of death in all age groups. Among the youngest, malaria (22.9% of all deaths in children under the age of 5 years; 16.2 and 4.8 deaths per 1,000 person-years in children aged <1 and 1–4 years) and acute respiratory infections (13.1% of all deaths in children aged <5 years; 21.2 and 1.5 deaths per 1,000 person-years in children aged <1 and 1–4 years, respectively) are responsible for the greatest losses. HIV/AIDS is the predominant cause of death of people aged 15–49 (11.6% of all deaths, corresponding to 0.6 deaths per 1,000 person-years in this age group). In contrast, deaths due to tuberculosis occurred mainly in those older than 15 years, peaking in the ≥65-year-old age group with 4.8 deaths per 1,000 person-years.

### Mortality due to non-communicable diseases by sex and age group

Non-communicable diseases accounted for 18.9% of all deaths, with an overall mortality rate of 1.3 deaths per 1,000 person-years. Acute abdomen (5.3% of all examined deaths; 0.4 deaths per 1,000 person-years), unspecified cardiac disease (2.1% of all examined deaths; 0.1 deaths per 1,000 person-years), digestive neoplasms (1.8% of all examined deaths; 0.1 deaths per 1,000 person-years), severe malnutrition, and strokes (each accounting for 2.7% of all examined deaths; 0.2 deaths per 1,000 person-years) were identified as the most frequent causes of death among the non-communicable diseases (Table 4).

**Table 4:** Mortality due to non-communicable diseases by sex and age group in the Taabo HDSS, 2009–2011

	<1 year			1–4 years			5–14 years			15–49 years			50–64 years			≥65 years		
	
Cause of death	_a_D_x_	%	_a_M_x__(‰)_	_a_D_x_	%	_a_M_x__(‰)_	_a_D_x_	%	_a_M_x__(‰)_	_a_D_x_	%	_a_M_x__(‰)_	_a_D_x_	%	_a_M_x__(‰)_	_a_D_x_	%	_a_M_x__(‰)_
	Male																	
Non-communicable diseases*	8	1.1	10.8	7	1.0	1.1	3	0.4	0.2	17	2.4	0.7	15	2.1	4.0	34	4.7	21.1
Acute abdomen	2	0.3	2.7	3	0.4	0.4	1	0.1	0.1	7	1.0	0.3	5	0.8	1.4	4	0.6	2.6
Acute cardiac disease	0	0.0	0.0	0	0.0	0.0	0	0.0	0.0	0	0.0	0.0	0	0.0	0.0	0	0.0	0.0
Asthma	0	0.0	0.0	0	0.0	0.0	0	0.0	0.0	0	0.0	0.0	0	0.0	0.0	0	0.0	0.0
Breast neoplasms	0	0.0	0.0	0	0.0	0.0	0	0.0	0.0	0	0.0	0.0	0	0.0	0.0	0	0.0	0.0
Chronic obstructive pulmonary disease	0	0.0	0.0	0	0.0	0.0	0	0.0	0.0	1	0.1	0.0	0	0.0	0.0	0	0.0	0.0
Congenital malformation	3	0.5	4.9	1	0.1	0.1	0	0.0	0.0	0	0.0	0.0	0	0.0	0.0	0	0.0	0.0
Diabetes mellitus	0	0.0	0.0	0	0.0	0.0	0	0.0	0.0	0	0.0	0.0	0	0.0	0.0	1	0.1	0.5
Digestive neoplasms	0	0.0	0.0	0	0.0	0.0	0	0.0	0.0	3	0.4	0.1	4	0.5	1.0	2	0.3	1.5
Epilepsy	0	0.0	0.0	1	0.1	0.1	2	0.2	0.1	2	0.2	0.1	0	0.0	0.0	0	0.0	0.0
Liver cirrhosis	0	0.0	0.0	0	0.0	0.0	0	0.0	0.0	0	0.0	0.0	0	0.0	0.0	0	0.0	0.0
Oral neoplasms	0	0.0	0.0	0	0.0	0.0	0	0.0	0.0	2	0.3	0.1	0	0.0	0.0	2	0.3	1.5
Other and unspecified cardiac disease	0	0.0	0.0	0	0.0	0.0	0	0.0	0.0	0	0.0	0.0	2	0.3	0.5	9	1.3	5.6
Other and unspecified non- communicable diseases	0	0.0	0.0	0	0.0	0.0	0	0.0	0.0	0	0.0	0.0	1	0.1	0.2	1	0.1	0.6
Other and unspecified neoplasms	0	0.0	0.0	0	0.0	0.0	0	0.0	0.0	1	0.2	0.0	1	0.1	0.3	4	0.5	2.2
Renal failure	0	0.0	0.0	0	0.0	0.0	0	0.0	0.0	0	0.0	0.0	0	0.0	0.0	1	0.1	0.5
Reproductive neoplasms	0	0.0	0.0	0	0.0	0.0	0	0.0	0.0	0	0.0	0.0	0	0.0	0.0	2	0.3	1.1
Respiratory neoplasms	0	0.0	0.0	0	0.0	0.0	0	0.0	0.0	1	0.2	0.0	0	0.0	0.0	1	0.1	0.4
Severe anemia	0	0.0	0.0	1	0.1	0.1	0	0.0	0.0	0	0.0	0.0	0	0.0	0.0	2	0.3	1.2
Severe malnutrition	2	0.3	3.2	2	0.3	0.3	0	0.0	0.0	0	0.0	0.0	0	0.0	0.0	1	0.1	0.4
Stroke	0	0.0	0.0	0	0.0	0.0	0	0.0	0.0	0	0.0	0.0	2	0.3	0.5	5	0.6	2.9
Sickle cell with crisis	0	0.0	0.0	0	0.0	0.0	0	0.0	0.0	0	0.0	0.0	0	0.0	0.0	0	0.0	0.0
	Female																	
Non-communicable diseases*	1	0.2	1.7	4	0.5	0.6	3	0.4	0.2	14	2.0	0.6	11	1.5	3.4	16	2.3	10.2
Acute abdomen	1	0.2	1.7	3	0.4	0.4	1	0.1	0.1	4	0.5	0.2	2	0.3	0.8	5	0.7	3.1
Acute cardiac disease	0	0.0	0.0	0	0.0	0.0	0	0.0	0.0	0	0.0	0.0	1	0.1	0.3	0	0.0	0.0
Asthma	0	0.0	0.0	0	0.0	0.0	0	0.0	0.0	0	0.0	0.0	0	0.0	0.0	0	0.0	0.0
Breast neoplasms	0	0.0	0.0	0	0.0	0.0	0	0.0	0.0	1	0.1	0.0	0	0.0	0.0	0	0.0	0.0
Chronic obstructive pulmonary disease	0	0.0	0.0	0	0.0	0.0	0	0.0	0.0	0	0.0	0.0	0	0.0	0.0	0	0.0	0.0
Congenital malformation	0	0.0	0.0	0	0.0	0.0	0	0.0	0.0	0	0.0	0.0	0	0.0	0.0	0	0.0	0.0
Diabetes mellitus	0	0.0	0.0	0	0.0	0.0	0	0.0	0.0	1	0.2	0.1	1	0.1	0.2	2	0.2	1.1
Digestive neoplasms	0	0.0	0.0	0	0.0	0.0	0	0.0	0.0	1	0.1	0.0	1	0.1	0.2	3	0.4	1.7
Epilepsy	0	0.0	0.0	0	0.0	0.0	0	0.0	0.0	1	0.1	0.0	0	0.0	0.0	0	0.0	0.0
Liver cirrhosis	0	0.0	0.0	0	0.0	0.0	0	0.0	0.0	1	0.1	0.0	1	0.2	0.4	1	0.1	0.5
Oral neoplasms	0	0.0	0.0	0	0.0	0.0	0	0.0	0.0	0	0.0	0.0	0	0.0	0.0	0	0.0	0.0
Other and unspecified cardiac disease	0	0.0	0.0	0	0.0	0.0	0	0.0	0.0	2	0.2	0.1	1	0.2	0.4	1	0.1	0.5
Other and unspecified non- communicable diseases	0	0.0	0.0	0	0.0	0.0	0	0.0	0.0	0	0.0	0.0	1	0.2	0.4	1	0.1	0.6
Other and unspecified neoplasms	0	0.0	0.0	0	0.0	0.0	0	0.0	0.0	0	0.0	0.0	0	0.0	0.0	2	0.3	1.2
Renal failure	0	0.0	0.0	0	0.0	0.0	0	0.0	0.0	1	0.1	0.0	0	0.0	0.0	0	0.0	0.0
Reproductive neoplasms	0	0.0	0.0	0	0.0	0.0	0	0.0	0.0	0	0.0	0.0	2	0.2	0.5	0	0.0	0.0
Respiratory neoplasms	0	0.0	0.0	0	0.0	0.0	0	0.0	0.0	1	0.1	0.0	0	0.0	0.0	0	0.0	0.0
Severe anemia	0	0.0	0.0	0	0.0	0.0	0	0.0	0.0	1	0.1	0.0	1	0.1	0.3	0	0.0	0.0
Severe malnutrition	0	0.0	0.0	1	0.1	0.1	2	0.3	0.1	0	0.0	0.0	0	0.0	0.0	2	0.3	1.3
Stroke	0	0.0	0.0	0	0.0	0.0	0	0.0	0.0	2	0.3	0.1	0	0.0	0.0	0	0.0	0.0
Sickle cell with crisis	0	0.0	0.0	1	0.1	0.1	0	0.0	0.0	0	0.0	0.0	0	0.0	0.0	0	0.0	0.0

_a_D_x_=cause-specific number of deaths in the sex-specific cohort from age a to age x.

% = percentage of all 712 cases of death that could be examined with the InterVA-4 tool.

_a_M_x (‰)_=cause-specific mortality rate per 1,000 person-years in the sex-specific cohort from age a to age x.

* Idiosyncrasies of InterVA-4 lead to minor rounding errors and explain small differences between the disaggregated data and the summed up totals as presented in this study.

In general, non-communicable diseases as cause of death were more common in males than females (0.8 *vs*. 0.5 deaths per 1,000 male and female person-years). The mortality rates due to severe malnutrition were highest during infancy (1.6 deaths per 1,000 person-years), whereas unspecified cardiac disease and stroke peaked in the oldest age group of 65 years and above (3.1 and 0.8 deaths per 1,000 person-years, respectively). All neoplasms together accounted for a total mortality rate of 0.3 deaths per 1,000 person-years with an increase of age-specific mortality rates with age (4.9 deaths per 1,000 person-years in people aged ≥65 years). Digestive neoplasms were particularly prominent neoplasms in both sexes in the oldest age group (1.5 and 1.7 deaths per 1,000 person-years in males and females, respectively), whereas oral neoplasms were prominent in the oldest age group among males (1.5 deaths per 1,000 person-years).

### Mortality due to maternal and neonatal conditions by sex and age group

Overall, 3,639 live births were registered between 2009 and 2011 in the Taabo HDSS (1,808 boys and 1,831 girls). In the same period and based on the data presented here, the overall neonatal mortality rate (defined as the number of all cause-unspecific deaths of newborns within the first 28 days after birth per 1,000 live births) was 16.7 deaths per 1,000 live births. The overall infant mortality rate (defined as the number of all cause-unspecific deaths of newborns within the first year after birth per 1,000 live births) was 34.0 deaths per 1,000 live births. Deaths due to typical maternal and neonatal conditions accounted for 8.3% of all analyzed deaths, resulting in an overall mortality rate of 0.6 deaths per 1,000 person-years (Table 5). The two main and typically neonatal conditions were neonatal pneumonia (2.6% of all examined deaths; 0.2 deaths per 1,000 person-years) and birth asphyxia (2.2% of all examined deaths; 0.2 deaths per 1,000 person-years). The two main causes of maternal death linked with pregnancy were obstetric hemorrhage (0.4% of all examined deaths; <0.1 deaths per 1,000 person-years) and pregnancy-induced hypertension (0.3% of all examined deaths; <0.1 deaths per 1,000 person-years). Overall, 34.2% of all examined deaths in children <1 year old were caused by typical neonatal conditions and 2.1% of all female deaths were caused by typical maternal conditions. In general, male newborns had a somewhat higher rate of death from neonatal conditions (19.2 male *vs*. 16.4 female deaths per 1,000 person-years).

**Table 5:** Mortality due to maternal and neonatal conditions by sex and age group in the Taabo HDSS, 2009–2011

	<1 year								
				
	Male			Female			Female (15–49 years)		
	
Cause of death	_a_D_x_	%	_a_M_x__(‰)_	_a_D_x_	%	_a_M_x__(‰)_	_a_D_x_	%	_a_M_x__(‰)_
Maternal/neonatal*	27	3.9	38.9	23	3.2	32.7	7	1.0	0.3
Anemia of pregnancy	0	0.0	0.0	0	0.0	0.0	1	0.1	0.0
Birth asphyxia	10	1.4	14.2	6	0.9	8.8	0	0.0	0.0
Congenital malformation	3	0.4	3.8	1	0.1	1.2	0	0.0	0.0
Neonatal pneumonia	11	1.5	15.5	8	1.1	11.1	0	0.0	0.0
Neonatal sepsis	0	0.1	0.7	2	0.3	3.2	0	0.0	0.0
Obstetric hemorrhage	0	0.0	0.0	0	0.0	0.0	3	0.4	0.1
Obstructed labor	0	0.0	0.0	0	0.0	0.0	0	0.0	0.0
Other and unspecified maternal cause of death	0	0.0	0.0	0	0.0	0.0	1	0.1	0.0
Other and unspecified neonatal cause of death	3	0.4	3.7	1	0.1	1.4	0	0.0	0.0
Pregnancy-induced hypertension	0	0.0	0.0	0	0.0	0.0	2	0.3	0.1
Pregnancy-related sepsis	0	0.0	0.0	0	0.0	0.0	1	0.1	0.0
Prematurity	1	0.2	1.8	5	0.7	7.0	0	0.0	0.0

_a_D_x_=cause-specific number of deaths in the sex-specific cohort from age a to age x.

% = percentage of all 712 cases of death that could be examined with the InterVA-4 tool.

_a_M_x (‰)_=cause-specific mortality rate per 1,000 person-years in the sex-specific cohort from age a to age x.

* Idiosyncrasies of InterVA-4 lead to minor rounding errors and explain small differences between the disaggregated data and the summed up totals as presented in this study.

### Mortality due to trauma and injury by sex and age group

Trauma and injury accounted for 3.8% of the examined deaths with an overall mortality rate of 0.3 deaths per 1,000 person-years. As summarized in Table 6, the most important sources of fatal trauma and injury were accidental drowning, contact with venomous plants or animals, traffic accident, and assault, together responsible for 2.4% of all deaths and an overall mortality rate of 0.2 deaths per 1,000 person-years.

**Table 6:** Mortality due to trauma and injury by sex and age group in the Taabo HDSS, 2009–2011

	<1 year			1–4 years			5–14 years			15–49 years			50–64 years			≥65 years		
	
Cause of death	_a_D_x_	%	_a_M_x__(‰)_	_a_D_x_	%	_a_M_x__(‰)_	_a_D_x_	%	_a_M_x__(‰)_	_a_D_x_	%	_a_M_x__(‰)_	_a_D_x_	%	_a_M_x__(‰)_	_a_D_x_	%	_a_M_x__(‰)_
	Male																	
Trauma and injury*	0	0.0	0.0	0	0.0	0.0	5	0.7	0.3	14	2.0	0.6	0	0.0	0.0	2	0.3	1.3
Accidental burns and smoke inhalation	0	0.0	0.0	0	0.0	0.0	0	0.0	0.0	1	0.1	0.0	0	0.0	0.0	0	0.0	0.0
Accidental drowning and submersion	0	0.0	0.0	0	0.0	0.0	1	0.1	0.1	2	0.3	0.1	0	0.0	0.0	0	0.0	0.0
Accidental fall	0	0.0	0.0	0	0.0	0.0	0	0.0	0.0	2	0.2	0.1	0	0.0	0.0	0	0.0	0.0
Accidental poisoning and noxious substances	0	0.0	0.0	0	0.0	0.0	0	0.0	0.0	0	0.1	0.0	0	0.0	0.0	0	0.0	0.0
Assault	0	0.0	0.0	0	0.0	0.0	1	0.1	0.1	4	0.5	0.1	0	0.0	0.0	1	0.1	0.6
Contact with venomous plants/animals	0	0.0	0.0	0	0.0	0.0	0	0.0	0.0	2	0.3	0.1	0	0.0	0.0	0	0.0	0.0
Intentional self-harm	0	0.0	0.0	0	0.0	0.0	1	0.1	0.1	1	0.1	0.0	0	0.0	0.0	1	0.1	0.4
Other and unspecified external cause of death	0	0.0	0.0	0	0.0	0.0	0	0.0	0.0	1	0.1	0.0	0	0.0	0.0	0	0.0	0.0
Traffic accident	0	0.0	0.0	0	0.0	0.0	2	0.3	0.1	2	0.3	0.1	0	0.0	0.0	0	0.0	0.0
	Female																	
Trauma and injury*	0	0.0	0.0	3	0.4	0.5	2	0.3	0.1	0	0.0	0.0	0	0.0	0.0	2	0.3	1.3
Accidental burns and smoke inhalation	0	0.0	0.0	1	0.1	0.2	0	0.0	0.0	0	0.0	0.0	0	0.0	0.0	0	0.0	0.0
Accidental drowning and submersion	0	0.0	0.0	0	0.0	0.0	1	0.1	0.1	0	0.0	0.0	0	0.0	0.0	0	0.0	0.0
Accidental fall	0	0.0	0.0	1	0.1	0.2	0	0.0	0.0	0	0.0	0.0	0	0.0	0.0	1	0.1	0.5
Accidental poisoning and noxious substances	0	0.0	0.0	0	0.0	0.0	0	0.0	0.0	0	0.0	0.0	0	0.0	0.0	0	0.0	0.0
Assault	0	0.0	0.0	0	0.0	0.0	0	0.0	0.0	0	0.0	0.0	0	0.0	0.0	0	0.0	0.0
Contact with venomous plants/animals	0	0.0	0.0	1	0.1	0.2	1	0.1	0.1	0	0.0	0.0	0	0.0	0.0	0	0.0	0.0
Intentional self-harm	0	0.0	0.0	0	0.0	0.0	0	0.0	0.0	0	0.0	0.0	0	0.0	0.0	0	0.0	0.0
Other and unspecified external cause of death	0	0.0	0.0	0	0.0	0.0	0	0.0	0.0	0	0.0	0.0	0	0.0	0.0	1	0.1	0.4
Traffic accident	0	0.0	0.0	0	0.0	0.0	0	0.0	0.0	0	0.0	0.0	0	0.0	0.0	0	0.0	0.0

_a_D_x_=cause-specific number of deaths in the sex-specific cohort from age a to age x.

%=percentage of all 712 cases of death that could be examined with the InterVA-4 tool.

_a_M_x (‰)_=cause-specific mortality rate per 1,000 person-years in the sex-specific cohort from age a to age x.

* Idiosyncrasies of InterVA-4 lead to minor rounding errors and explain small differences between the disaggregated data and the summed up totals as presented in this study.

Males were twice as likely to die from trauma or injury as females (0.2 male *vs*. 0.1 female deaths per 1,000 person-years). Most notably, all victims of assault (*n*=6) and traffic-related accidents (*n*=4) were male. Whereas 14 men aged 15–49 years died of trauma or injury, there were no female deaths in this age group resulting from these causes. The age-specific mortality rates for trauma and injury were highest in the 15–49 years age group (0.3 deaths per 1,000 person-years), followed by the 1–4, 5–14, and ≥65 years age groups (all 0.3 deaths per 1,000 person-years). No fatalities due to trauma or injury were reported for the <1 year and the 50–64 years age groups.

### Mortality due to causes that could not be determined despite complete VA data

For 10.0% of the deaths with complete VA data, no specific cause could be determined (0.7 deaths per 1,000 person-years) (Table 7). There was no gender difference in mortality due to undetermined causes of death. The highest percentages of deaths with undetermined causes despite complete VA data were in the 15–49 and ≥65 years age groups, with 2.3 and 2.1%, respectively. In the 5–14 years age group, the rate of undetermined death was lowest, with 0.5%.

**Table 7:** Mortality due to undetermined causes by sex and age group in the Taabo HDSS, 2009–2011

	Male			Female			Total*		
	
Age (years)	_a_D_x_	%	_a_M_x__(‰)_	_a_D_x_	%	_a_M_x__(‰)_	_a_D_x_	%	_a_M_x__(‰)_
<1	8	1.0	10.7	6	0.8	8.8	14	1.9	9.7
1–4	5	0.7	0.8	5	0.8	0.8	11	1.5	0.8
5–14	2	0.2	0.1	2	0.3	0.2	4	0.5	0.1
15–49	9	1.3	0.4	7	1.0	0.3	16	2.3	0.3
50–64	7	0.9	1.7	5	0.7	1.7	12	1.7	1.7
≥65	8	1.1	4.9	7	0.9	4.5	15	2.1	4.7
All*	38	5.3	0.7	33	4.7	0.7	71	10.0	0.7

This table summarizes the mortality due to causes that could not be determined despite complete data from verbal autopsy.

_a_D_x_=cause-specific number of deaths in the sex-specific cohort from age a to age x.

% = percentage of all 712 cases of death that could be examined with the InterVA-4 tool.

_a_M_x (‰)_=cause-specific mortality rate per 1,000 person-years in the sex-specific cohort from age a to age x.

* Idiosyncrasies of InterVA-4 lead to minor rounding errors and explain small differences between the disaggregated data and the summed up totals as presented in this study.

## Discussion

Recognizing the paucity of vital statistics in Côte d'Ivoire, particularly after a decade-long political and military crisis that further deteriorated an already fragile health system (31), we analyzed VA data from the first 3 years of operation of the Taabo HDSS (2009–2011) to identify sex- and age-specific mortality rates and causes of death. The top five causes of death that we identified in the Taabo HDSS were malaria (18.0% of the 712 analyzed deaths), respiratory tract infections (15.4%), HIV/AIDS (11.2%), pulmonary tuberculosis (6.5%), and acute abdomen (5.3%).

### Comparison with other data from Côte d'Ivoire on causes of death

Comparing our results with other readily available data from Côte d'Ivoire on causes of death presents the difficulty of comparing predominantly rural data (e.g. Taabo HDSS, presented here) with overall country data and with data potentially derived through different forms of data collection and with subtle differences in definitions. Hence, we used mortality data from the World Health Statistics 2014 and the most recent MICS, which we considered as suitable reference points despite the aforementioned idiosyncrasies (Table 8). The World Health Statistics 2014 contains national estimates for the year 2012 and the MICS data were collected nationwide in 2011 and 2012 (8, 9).

**Table 8:** Comparison of the Taabo HDSS mortality data (2009–2011) with other estimates for Côte d'Ivoire

	Taabo HDSS	National comparison data
Cause-specific mortality (mortality rate provided per 1,000 person-years in total population)^a^		
Communicable disease^b^	4.0	8.6*
Malaria	1.2	0.7*
HIV/AIDS-related	0.8	1.6*
Non-communicable disease	1.3	7.9*
Trauma and injury	0.3	1.2*
Adult mortality (all-cause mortality rate provided per 1,000 person-years in adults 15–49 years)		
Crude male mortality	5.4	4.9#
Crude female mortality	4.8	5.8#
Reproductive health (mortality rate provided per 1,000 live births)		
Maternal mortality	2.5	6.1#
Infant mortality^c^	34.0	68.0#
Under-five mortality^d^	94.3	108.0#
Cause-specific mortality in children under 5 years (distribution of causes of deaths in %)		
Malaria	29.7	16.0*
Acute respiratory infections and pneumonia	16.7	15.0*
HIV/AIDS	10.2	2.0*
Diarrhea-related	5.5	10.0*
Intrapartum-related/birth asphyxia	5.5	12.0*
Congenital anomalies	2.7	5.0*
Prematurity	2.0	13.0*
Trauma and injury	1.0	5.0*
Neonatal sepsis	0.7	8.0*
Neonatal pneumonia	6.5	–
Measles	0.3	0.0*
Other conditions	18.8	14.0*

* Comparison data marked with are from the World Health Statistics 2014, which provides data for 2012 (8).

# Comparison data marked with are from the most recent Multiple Indicator Cluster Survey, which provides data collected in late 2011 and early 2012 (9).

a Originally, the comparison data were age-standardized and provided as per 100,000 population. For comparison, the data were transformed into crude mortality rates per 1,000 person-years by assuming 1,000 population = 1,000 person-years and ignoring any age standardization.

b The cluster of communicable diseases consists of all infectious, contagious, maternal, neonatal, and nutritional conditions, even though the latter three conditions are not communicable. These conditions were grouped together to facilitate comparison with the World Health Statistics 2014, which also uses this grouping (8).

c Defined as all-cause mortality in children under 1 year.

d Defined as all-cause mortality in children under 5 years.

As revealed by Table 8, our results from the Taabo HDSS suggest (except for males) generally lower mortality rates compared to the World Health Statistics and MICS data for all of Côte d'Ivoire (8, 9). These lower rates might be due to slight differences in indicator definitions and to the fact that most Taabo HDSS indicators included only deaths with unambiguously defined cause by means of VA. However, the pattern we encountered, indicating communicable diseases as the most frequent cause of death, followed by non-communicable diseases and then trauma and injury, is congruent. Of note, overall mortality rates due to malaria are higher in the Taabo HDSS than the national average estimates, which is not surprising as the area is well-known to be highly malaria endemic (32), whereas the HIV/AIDS-related mortality seems to be considerably lower. The importance of malaria in the Taabo HDSS is further underscored by the fact that almost every third death in children under 5 years of age is attributable to this disease. With regard to HIV/AIDS, it is noteworthy that children under five seem to be disproportionally affected. Another striking feature of the under-five mortality estimates from the Taabo HDSS is the comparatively low number of deaths due to premature birth. In general, the Taabo HDSS estimates for overall maternal, infant, and under-five mortality are lower than other data suggest for the whole of Côte d'Ivoire. Finally, it may be interesting to point out that, although a WHO report on the environmental burden of disease for Côte d'Ivoire considers disability-adjusted life years for asthma to be the most important environmental burden (33), we did not have a single asthma death and there was only one chronic obstructive pulmonary disease death reported (Table 4).

### Comparison with international low- and middle-income country data

The comparison with mortality data from other settings in low- and middle-income countries allows us to put our findings into a broader context. Contrary to Taabo, for example, the most striking feature of the structure of causes of death in Agincourt, South Africa, is the high mortality from accidents and violent deaths (29). Furthermore, in the South African setting, malaria is of lesser importance, but HIV/AIDS, tuberculosis, and dysentery and diarrhea are leading causes of death, largely explained by the HIV epidemic. For further comparison of our findings with those of other HDSS sites, the reader is referred to a collection of articles published in a recent issue of *Global Health Action* (14–19). In summary, as in the Taabo HDSS and many other low-income settings in sub-Saharan Africa, one still notes a predominance of death due to infectious disease and mother and child health issues, which is typical for tropical countries with weak health-care performance (34). The Taabo HDSS estimates for overall maternal, infant, and under-five mortality, which seem relatively low when compared with other data sources for Côte d'Ivoire (Table 8), are in fact well within the range of the estimates from other HDSS sites (14, 17).

### Public health relevance for rural Côte d'Ivoire

Our age- and sex-specific mortality rates provide a benchmark for the current epidemiological situation in a typical rural Ivorian setting and, hence, offer useful information for health services and potential public health actions to be taken. Overall, 375 of 948 recorded deaths (39.6%, Table 1) occurred in children before they reached their fifth birthday. Furthermore, 79.2% of the deaths that we were able to analyze in full detail in this age group were attributed to infections (communicable diseases and neonatal pneumonia and sepsis), for most of which preventive or curative measures exist. This indicates a substantial potential for public health interventions to effectively lower mortality rates. Furthermore, with 50 of 149 examined deaths in children below the age of 1 year (33.6%) occurring due to neonatal causes and 8 of 78 examined deaths in women aged between 15 and 49 years (10.2%) attributed to maternity complications, the importance of improved maternal and newborn care is evident.

HIV/AIDS was identified as the third most important cause of death in this rural part of south-central Côte d'Ivoire. In adolescents and young adults (aged 15–49 years), 16.8% of deaths analyzed were attributable to HIV/AIDS and another 12.2% possibly to HIV/AIDS-related pulmonary tuberculosis. These figures, together with the high rates of death attributed to HIV/AIDS in children under five (10.2% of all analyzed under-five deaths), clearly indicate the need for further HIV prevention and case management and particularly also for preventive measures to avoid mother-to-child transmission.

The clinical presentation acute abdomen is caused by a variety of conditions usually presenting with severe abdominal pain. Many of these patients can be saved by an emergency operation or other supportive care. This outcome requires prompt recognition of the disease severity, often through a simple physical examination, and usually referral to the district hospital level for operation or care. In the Taabo HDSS setting, roughly about every twentieth death falls into this category. Further investigation into why these people were not saved is warranted. Concerning the predominance of male deaths due to acute abdomen, the role of incarcerated hernia or the role of alcohol-induced acute pancreatitis should be investigated; another factor that deserves research is the possibility of delayed health-care seeking that might be gender-related in terms of knowledge, attitudes, practices, and beliefs on how to deal with pain. Furthermore, in men aged 15–49 years, trauma and injury, and most notably assault, are important causes of death (all together 14.9% of the male deaths examined in this age group), while in the same age group no women died of a similar cause. This fact may indicate that in addition to professional exposure, behavioral and social factors are important.

### Limitations

As with any epidemiological and demographic survey, correct estimation of the reference population is a critical issue. Although we cannot completely rule out a certain imprecision in the reference population, the population database of the Taabo HDSS exhibits a high validity and reliability. Indeed, it is based on standard procedures implemented at HDSS sites and relies on regular visits to all households under surveillance once every 3–6 months (13).

Another source of imprecision may stem from the VA interviews. VA relies on information provided by close family members and their answers might be subject to recall or cultural bias. Hence, we cannot rule out the possibility that certain causes of death are over- or under-reported. However, because VA interviews usually take place shortly after a case of death occurs (usually within 2–4 weeks) and because the loss of a close person is a dramatic event, the risk of recall bias may be comparatively low. Furthermore, VA questions were neutrally formulated and administered by specifically trained Ivorian field workers in order to minimize any potentially occurring cultural bias.

It is likely that certain health services and behavioral factors influenced our findings in one or the other way. For instance, in the case of HIV/AIDS and pulmonary tuberculosis, we note higher rates of HIV/AIDS in women and higher rates of pulmonary tuberculosis in men. If we add up the deaths due to these two infectious diseases, which are often related, we find very similar overall rates for both sexes (3.6 and 3.5% of all analyzed deaths). Although transmission rates of HIV after exposure during sexual intercourse are higher for women (35), potentially explaining the higher HIV death toll in females, results may also be influenced by factors such as sero-status known through HIV testing being proposed to pregnant women, but not to prospective fathers. By contrast, alcohol consumption is a risk factor for tuberculosis (36) and is more prevalent among men than women in the Taabo HDSS.

In a setting with stronger health services, older individuals may have better knowledge of their underlying conditions, such as hypertension or diabetes, or certain neoplasms – conditions that warrant measures other than just interviewing for diagnosis. With further strengthening of the health system, such information could become increasingly available and also support the determination of causes of death, thereby potentially increasing the number of confirmed cardiovascular- and cancer-related deaths and reducing the number of undetermined deaths. Still, the four reported strokes in adults below the age of 65 years alert us to the fact that chronic cardiovascular disease may be of greater importance in rural Africa than is currently assumed.

## Conclusions

The data presented here are another confirmation that death due to communicable diseases and maternal and early childhood conditions are still rampant in rural settings of sub-Saharan Africa. However, the data also reveal that death due to chronic diseases already affects young people. Many deaths occur due to afflictions that could be either prevented or treated, indicating the urgent need for public health action and health service strengthening.

Despite the still relatively small number of deaths recorded and analyzed in the Taabo HDSS, the presented work provides a useful benchmark for the years to come. It can assist in studying demographic and epidemiological transitions that are already underway in many settings of Côte d'Ivoire and elsewhere in West Africa. Hence, our data can serve as a ‘historical control’ and for promoting and determining the impact of specific public health interventions. The Taabo HDSS and the INDEPTH Network more broadly are platforms that will contribute by responding to some of the most pressing public health questions, such as ‘Where and why are 10 million children dying every year?’ (37). Moreover, progress toward the Millennium Development Goals can be measured with high fidelity (38) and we are convinced that these platforms will be of central relevance in the post-2015 agenda of sustainable development. Hence, vital statistics must be seen as an indispensable part of any development infrastructure with social, political, and economic benefits that go far beyond the health sector (39). It is our hope that the work presented here will shape setting-specific public health actions to improve the health and well-being in the Taabo region and far beyond.
